# Do “auditory” and “visual” time really feel the same? Effects of stimulus modality on duration and passage-of-time judgements

**DOI:** 10.3758/s13414-025-03131-5

**Published:** 2025-07-25

**Authors:** Daniel Bratzke

**Affiliations:** Department of Psychology, University of Bremen, Bremen, Germany

**Keywords:** Time perception, Passage of time, Modality

## Abstract

The present study investigated the previous claim that auditory stimuli appear to last longer than visual ones, but that the modality has no influence on the experience of the passage of time (POT). Participants judged the duration, the POT, and the phenomenal quality of the two temporal experiences after hearing a tone or viewing a blue square with a duration between 200 ms and 5 s. The results showed modality effects on both duration and POT judgements, with longer duration and slower POT judgements for auditory than for visual stimuli. Judgements of phenomenal quality showed large interindividual differences, with most participants showing positive but some also negative relationships with target duration for both qualities. Importantly, duration and POT judgements were largely unaffected by these interindividual differences. The present results clearly contradict the previous assumption that the experienced POT is not influenced by sensory modality.

## Introduction

It is a well-established finding that auditory stimuli are perceived as being longer than visual stimuli of the same duration (e.g., Goldstone & Goldfarb, 1964; Wearden et al., 1998). In 2015, Wearden stated that “auditory stimuli are judged as longer than visual ones by around 20%. But nothing, apart from the obvious sensory difference, ‘feels’ different about the duration experience. People do not think that time is flying or dragging in one modality compared with another one.” (p. 166). This claim is consistent with a growing body of research suggesting that the relationship between duration and passage of time (POT) judgements is less close than one would probably expect (e.g., Droit-Volet & Wearden, 2016; Droit-Volet et al., 2017; Jording et al., 2022; Ogden et al., 2011; Wearden, 2015), especially when relatively short durations of only a few seconds are considered (Martinelli & Droit-Volet, 2022a).

The difference between auditory and visual duration perception has often been investigated in the milliseconds to seconds range (e.g., Bratzke & Ulrich, 2019; Goldstone & Goldfarb, 1964; Wearden et al., 1998). So far, the relationship between duration and POT experience has essentially not been researched in the milliseconds range, probably also because it is sometimes assumed that an awareness of POT does not arise for relatively short durations (< 2–3 s; see Droit-Volet et al., 2017). One recent exception is the study by Bratzke and Hansen (2024), which investigated duration and POT judgements in a speeded reaction time (RT) task, and showed that the effects of task difficulty on RT were reflected in both duration and POT judgements. Even though this result pattern can be viewed as first evidence for a close relationship between duration and POT judgements in the milliseconds range, introspective judgements about RT performance may be qualitatively different from temporal judgements of external events (see Bratzke & Bryce, 2022; Klein & Stolz, 2018).

Another study that points to the possibility that Wearden’s above claim might have been too hasty is the study by Martinelli and Droit-Volet (2022b). These authors investigated what factors can affect prospective POT judgements (for a classification of POT judgements, see Droit-Volet & Martinelli, 2023) in simple laboratory tasks, including viewing neutral pictures of different duration (black and white geometric patterns; Exp. 3). Stimulus duration turned out to be one of the factors that influenced POT judgements, and this was the case in the seconds as well as in the minutes range. Overall, the authors concluded that POT judgements can be affected by a number of factors, including the perception of duration. Since the perception of duration differs between the auditory and the visual modality, one might therefore assume that the experience of POT would also differ between the two modalities.

It is commonly assumed that prospective timing (of duration) is based on an internal clock mechanism, where the perceived duration is represented by the number of pulses (elicited by a pacemaker) counted by an accumulator during a to-be-timed interval (e.g., Gibbon et al., 1984; Zakay & Block, 1995). Modality effects on duration judgements have often been explained within this framework by assuming that the pacemaker runs faster for auditory than for visual stimuli (e.g., Bratzke & Ulrich, 2019; Wearden et al., 1998). In contrast, POT judgements have been assumed to be based on the introspective analysis of internal states (e.g., emotions), which result from the effect of context on the minimal self (e.g., Droit-Volet & Martinelli, 2023; Martinelli & Droit-Volet, 2022b). The relationship between these two theoretical accounts is largely unclear. Martinelli and Droit-Volet (2022b) suggested that in prospective timing paradigms (i.e., when participants know in advance that they are asked for temporal judgements), POT judgements should be influenced by duration, as in this case the internal cock mechanism is mobilized. They also concluded that POT judgements can be multifaceted, and that one of these facets can be based on the processing of durations, which also opens the possibility for modality effects on POT judgements from a theoretical perspective.

The present study investigated duration and POT judgements in a prospective timing task similar to the one of Wearden et al. (1998). As in their study, participants were presented with auditory (500-Hz sine tone) and visual (blue square) stimuli of different durations. Similar to the verbal estimation in Wearden et al.’s Experiment 2, participants provided their judgements on visual analogue scales (VASs; see Bratzke & Hansen, 2024; Martinelli & Droit-Volet, 2022b). Additionally, since it has been suggested that an awareness of POT may not arise for relatively short durations (see Droit-Volet et al., 2017), the present study aimed to explore the duration at which an awareness of POT emerges. To this end, participants were also asked to indicate the degree to which they experienced the phenomenal quality of duration and POT, and the range of durations tested by Wearden et al. (77–1,183 ms) was extended into the seconds range (200–5,000 s).

### Method

#### Participants

Sixty-seven volunteers (59 female, four male, three nonbinary; mean age = 24.8 years, range 18–55 years, one participant did not report age) participated for course credit. A power analysis indicated that 52 participants would be sufficient to detect a two-way repeated-measures interaction with alpha =.05, power =.80 and a large effect size of η_p_^2^ =.14 (d_z_ =.40; see Langenberg et al., 2023). All participants provided informed consent prior to data collection in accordance with the 1964 Helsinki Declaration and its later amendments.

#### Apparatus and stimuli

The experiment was an online experiment and run on the participant’s individual computer. It was created in PsychoPy (Peirce et al., 2019) and hosted by Pavlovia (https://pavlovia.org). The PsychoPy code ScreenScale (Morys-Carter, 2021) was used to adjust the screen scale on individual computers. The auditory stimuli were 500-Hz sine tones with 5-ms rise and fall times. The visual stimulus was a blue square (4 × 4 cm), presented on a gray background. Temporal judgements were assessed using VASs (horizontally centered straight black lines covering about 56% of the screen width with small ticks at the left and right end). Each VAS was accompanied by a question above the VAS and labeled at the left and right end. The questions were “How long did the stimulus last?” (left label: “very short” vs. right label: “very long”) for the duration judgement, “How pronounced was the experience of duration?” (left label: “not at all” vs. right label: “very clearly”) for the duration quality judgement, “How quickly did time pass?” (left label: “very slowly” vs. right label: “very quickly”) for the POT judgement, and “How pronounced was the experience of time passing?” (left label: “not at all” vs. right label: “very clearly”) for the POT quality judgement. When clicking on the respective scale, a small black dot appeared at the indicated position.

#### Tasks and procedure

**Fig. 1:**
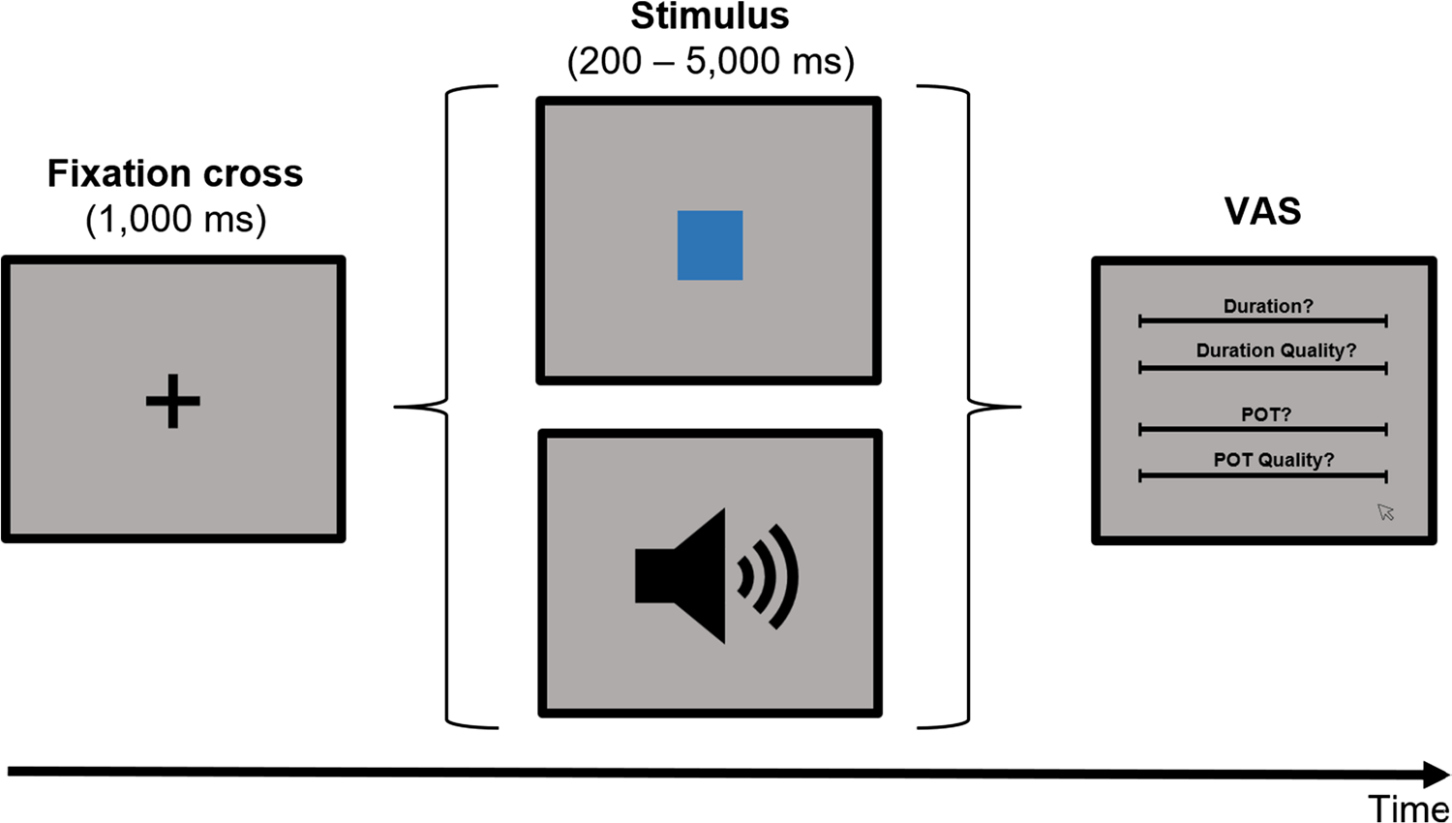
Schematic depiction of an experimental trial. First, a fixation cross appeared, followed by a blue square or a 500-Hz sine tone. At the end of each trial, participants were asked to provide judgements of duration, duration quality, passage of time (POT), and POT quality on separate visual analogue scales (VASs)

At the beginning of the experiment, participants were asked to adjust the screen scale and the sound level of their computer. For the sound level adjustment, a 500-Hz sine tone was presented until participants pressed a key to indicate that the sound level was at a comfortable level. In each trial of the temporal task (see Fig. 1), first a fixation cross appeared on the screen for 1,000 ms. Then, a tone or a blue square was presented for one of seven possible target durations (200, 1,000, 1,800, 2,600, 3,400, 4,200, or 5,000 ms). After the offset of the target stimuls, the four VASs appeared on the screen, in the order duration, duration quality, POT, and POT quality from top to bottom. Participants clicked on the VASs to provide their judgements and confirmed them with a keypress of the space key (only possible after all judgements were provided). Participants performed a total of 112 experimental trials (eight trials for each target duration and modality), divided into four blocks. The experiment lasted about 30 min.

## Results

Visual inspection of individual data revealed that all participants showed an effect of target duration on duration judgements in the expected direction, therefore no data were excluded from further analyses. There were also interindividual differences in the trend of quality judgements across target durations for both qualities. Although most participants showed positive trends, some showed negative or U-shaped functions. Therefore, as a first step the data were analyzed at the aggregated level, and interindividual differences were taken into account in a second step.

### Duration and passage-of-time (POT) judgements

**Fig. 2:**
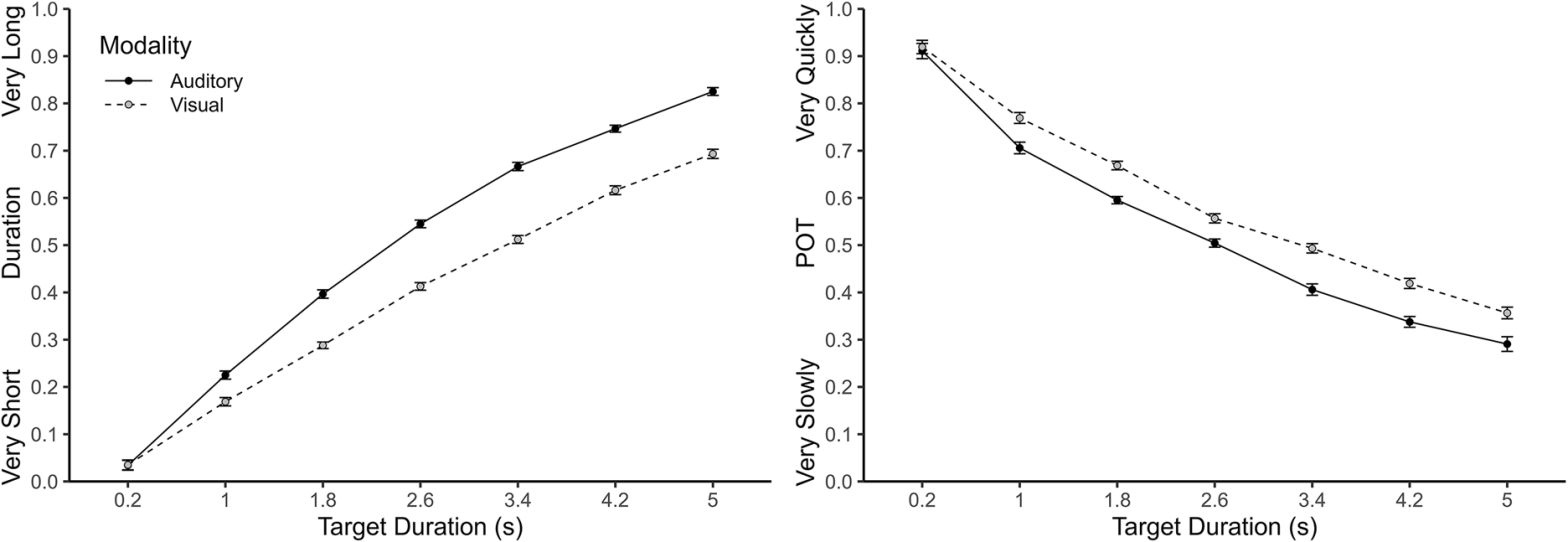
Duration and passage-of-time (POT) judgements as a function of target duration and modality. Error bars represent ± 1 within-subjects *SE* (Morey, 2008)

As can be seen in Fig. 2, duration and POT judgements showed opposing effects of target duration and therefore POT judgements were inverted for further analyses (see also Bratzke & Hansen, 2024). An overall ANOVA with the within-subject factors judgement type (duration vs. POT), modality (auditory vs. visual), and target duration was conducted on duration and inverted POT judgements. As expected, there were significant main effects of target duration, *F*(1, 66) = 939.76, *p* <.001, η_p_^2^ =.93, and modality, *F*(1, 66) = 199.78, *p* <.001, η_p_^2^ =.75. The main effect of judgement type was not significant, *F*(1, 66) = 0.42, *p* =.519, η_p_^2^ =.01. All two-way interactions and the three-way interaction were significant. The effect of modality increased with increasing target duration, *F*(6, 396) = 23.19, *p* <.001, η_p_^2^ =.26, and this pattern was more pronounced for duration than for POT judgements, *F*(6, 396) = 15.07, *p* <.001, η_p_^2^ =.19. The significant interaction between judgement type and modality, *F*(6, 396) = 27.13, *p* <.001, η_p_^2^ =.29, also indicated a larger effect of modality on duration (.10) than on POT (.06) judgements. Similarly, the significant interaction between judgement type and target duration, *F*(6, 396) = 38.40, *p* <.001, η_p_^2^ =.37, indicated that the effect of target duration was larger for duration (.72) than for POT (.59) judgements. The three-way interaction was also significant, *F*(6, 396) = 15.07, *p* <.001, η_p_^2^ =.19. As can be seen in Fig. 2, the interaction between target duration and modality was somewhat smaller for POT than for duration judgements. Crucially, separate ANOVAs showed significant main effects of modality and target duration and a significant interaction for both judgement types, all *p*s <.001.

To examine the relationship between duration and POT judgements, repeated-measures correlations (Bakdash & Marusich, 2017) were calculated separately for each target duration. This analysis revealed moderate to strong correlations between duration and inverted POT judgements (from .37 to .59; see also left panel of Fig. 4). An ANOVA on individual Pearson correlations between the two judgements showed no significant effect of target duration, *F*(6, 396) = 1.25, *p* =.288, η_p_^2^ =.02.

### Duration and POT quality judgements

**Fig. 3:**
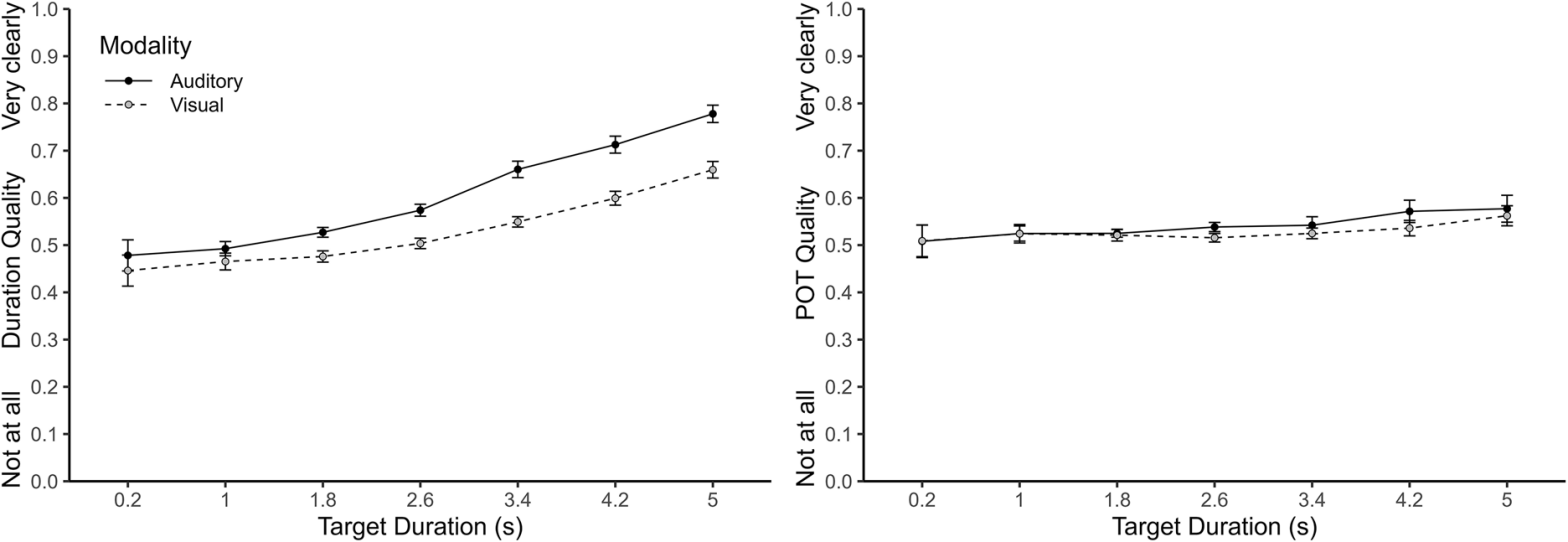
Duration and passage-of-time (POT) quality judgements as a function of target duration and modality. Error bars represent ± 1 within-subjects *SE* (Morey, 2008)

Judgements of phenomenal quality for both duration and POT started around .5 and increased with target duration in the case of duration quality but less so for POT quality (see Fig. 3). An ANOVA with the within-subject factors judgement type (duration vs. POT quality), modality (auditory vs. visual), and target duration revealed significant main effects of target duration, *F*(1, 66) = 17.74, *p* <.001, η_p_^2^ =.21, and modality, *F*(1, 66) = 71.84, *p* <.001, η_p_^2^ =.52, but no significant main effect of judgement type, *F*(1, 66) = 3.46, *p* =.067, η_p_^2^ =.05. The significant interaction between judgement type and modality, *F*(3, 396) = 15.37, *p* <.001, η_p_^2^ =.21, confirmed a larger effect of modality on duration (.07) than POT (.01) quality judgements. The significant interaction between judgement type and target duration, *F*(3, 396) = 8.81, *p* =.002, η_p_^2^ =.12, indicated that the increase of quality with increasing target duration was larger for duration (difference between longest and shortest duration: .26) than for POT (.06). The three-way interaction was also significant, *F*(6, 396) = 8.81, *p* =.002, η_p_^2^ =.12. Separate ANOVAs for duration and POT quality revealed that both main effects (modality and target duration) as well as the interaction were significant for duration quality, all *p*s <.001, whereas none of these effects was significant for POT quality, all *p*s ≥.174.

**Fig. 4:**
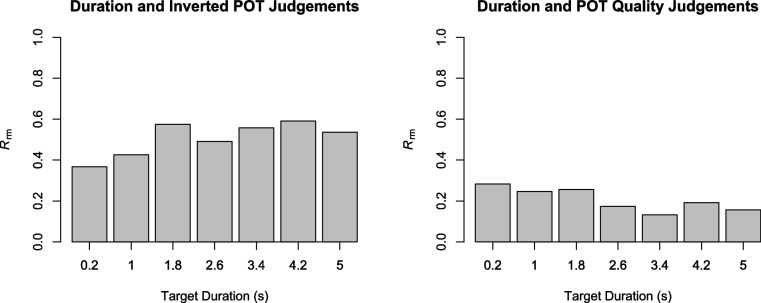
Repeated-measures correlations (*R*_rm_) between duration and inverted passage-of-time (POT) judgements, and between duration and POT quality judgements. All correlations are significant with *p* <.05

Correlational analyses showed weak but significant positive correlations between duration and POT quality judgements (from .13 to .28; see right panel of Fig. 4). An ANOVA on individual Pearson correlations between the two quality judgements showed no significant effect of target duration, *F*(6, 396) = 1.25, *p* =.288, η_p_^2^ =.02.

**Fig. 5:**
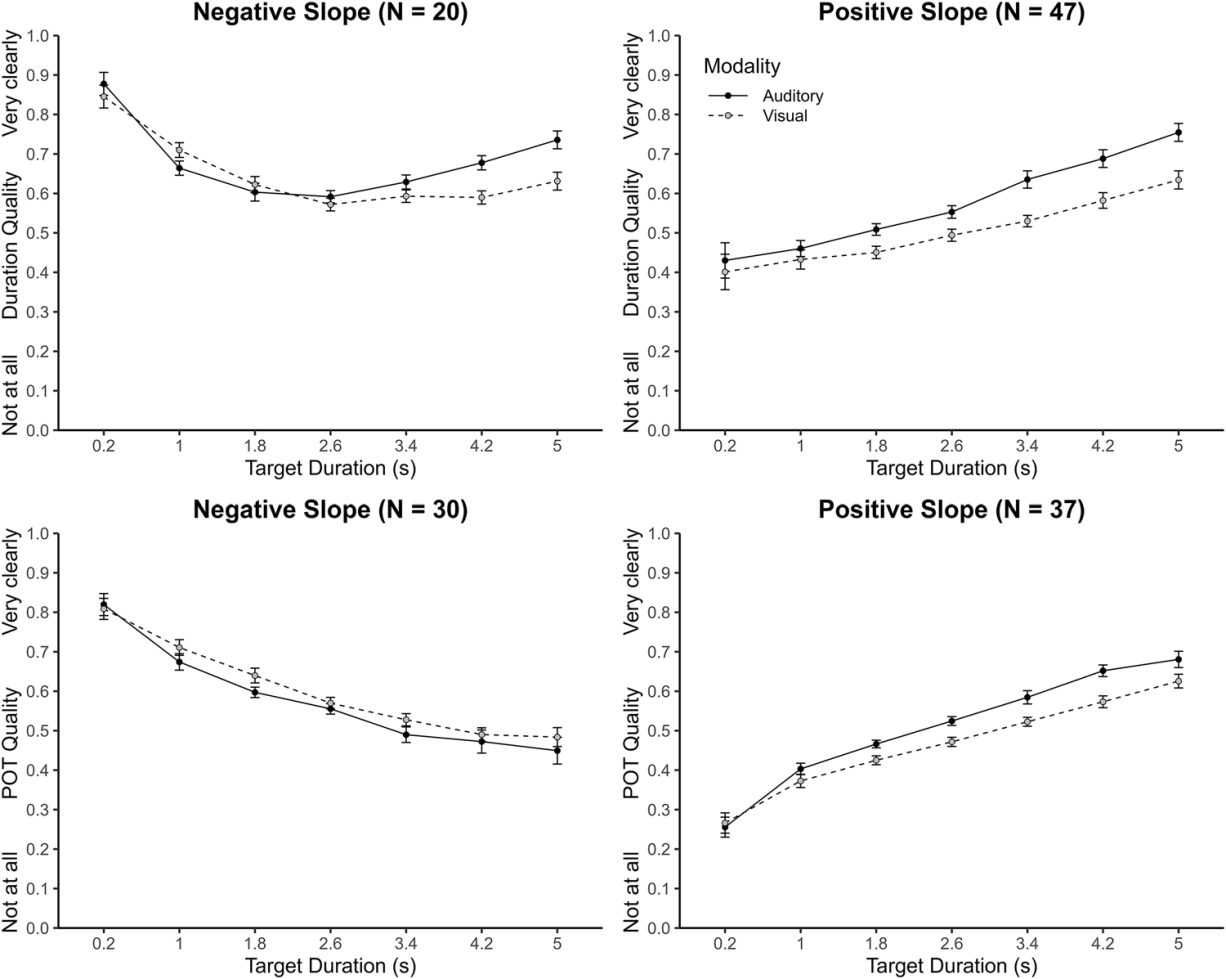
Duration and passage-of-time (POT) quality judgements as a function of group (negative vs. positive slope), target duration, and modality. Error bars represent ± 1 within-subjects *SE* (Morey, 2008)

To further explore interindividual differences in the trends of quality judgements across target durations, linear regression analyses were conducted on individual data with target duration as predictor and quality judgement as outcome variable. For duration quality, individual slopes ranged from -.11 to .17, with 20 participants showing negative and 47 participants showing positive slopes. For POT quality, the range of slopes was similar (-.18 to .18), but the proportion of participants with a negative and a positive slope was more balanced (30 negative and 37 positive). Only ten of the 30 participants who showed a negative slope for POT quality also showed a negative slope for duration. Figure 5 shows quality judgements as a function of target duration and modality for the different groups of participants (negative vs. positive slope). As can be seen in the upper panels, for duration quality the positive slope group showed a pattern very similar to the overall mean pattern (see Fig. 3), whereas the negative slope group showed a different pattern up to about 3 s. An ANOVA with the additional factor group indicated that all interactions were significant, all *p*s *≤*.006. Separate ANOVAs for the two groups showed that the interaction between modality and target duration was significant for both groups, *p*s ≤.001. For POT quality, the pattern clearly differed across the whole range of target durations (see lower panels of Fig. 5). An ANOVA with the additional factor group revealed a significant interaction between modality and target duration, *F*(1, 65) = 14.82, *p* <.001, η_p_^2^ =.19, and a significant three-way interaction, *F*(6, 390) = 4.09, *p* =.002, η_p_^2^ =.06. The interaction between modality and target duration was significant for the group with positive slopes (*p* =.001), but not for the group with negative slopes (*p* =.263).

### Reanalysis of duration and POT judgements

Importantly, reanalysing duration and POT judgements in two seperate ANOVAs with the additional factors duration group (negative vs. positive) and POT group (negative vs. positive) based on individual regression slopes for duration and POT quality judgements revealed that these group factors did not substantially alter the result pattern. The only significant effects related to the group factors were the main effect of duration group (*p* =.043), the interaction between duration group and modality (*p* =.020), and the interaction between POT group and judgement type (*p* =.021). Participants with positive duration quality slopes showed slightly larger temporal judgements (.45) than the ones with negative slopes (.40), and the effect of modality was slightly larger for participants with positive (.09) than negative (.06) slopes. With regard to POT quality, there was a difference between duration and POT judgements for the participants with negative slopes (.11), wheras this difference was virtually zero (<.01) for the ones with positive slopes.

## Discussion

The present study investigated the experience of the POT between the auditory and visual modality. While it has long been known that auditory stimuli are judged as being longer than visual ones, empirical evidence regarding the feeling of how time passes with these stimuli was essentially missing. The present results show that auditory stimuli are not only judged as being longer than visual ones but time is also judged as passing more slowly in the presence of auditory compared to visual stimuli. This clearly suggests that the previous claim that “…nothing ‘feels’ different about the duration experience…in one modality compared with another one.” (Wearden, 2015, p. 166) was too hasty.

The factor sensory modality adds to a number of previously established non-temporal factors that can affect POT judgements, such as task difficulty and emotional valence (Martinelli & Droit-Volet, 2022a, b). Martinelli and Droit-Volet (2022b) suggested that which of these factors becomes effective should depend on the saliency of the factors in the encountered context. Accordingly, sensory modality might have affected POT judgements in the present study because the modality differences as well as their associated effects on duration judgements were especially salient. Future studies could investigate whether the effect of sensory modality on POT can be modulated by the addition of other factors, such as emotional valence, and whether such potential modulations would be accompanied by similar modulations of duration judgements or not. This could provide further insights into the question of whether duration and POT judgements are based on the same or different underlying timing mechanisms (see Bratzke & Hansen, 2024; Martinelli & Droit-Volet, 2022a).

The present results clearly show that modality affects both the perceived duration and the feeling of how time passes. Nevertheless, there were also differences between the effects of modality, and also target duration, on duration and POT judgements, with the effects being slightly larger for duration than for POT judgements. Although it is difficult to directly compare duration and POT judgements, as they presumably reflect different phenomenal experiences, these differential results might suggest that for the employed time range (a) POT judgements are not simply the inverse of duration judgements (consistent with the clearly far from perfect correlations between the two judgements), (b) differences in the experience of POT are less pronounced than differences in perceived duration, and (c) the experience of duration is generally more pronounced than the experience of POT (see also the overall somewhat higher judgements for duration than POT quality). Importantly, the present results and all their possible implications are of course limited to very simple stimuli in the time range of milliseconds to seconds and might differ for more complex stimuli and longer durations.

The results regarding phenomenal quality of POT (and duration) were rather surprising, as there were large interindividual differences, with some participants showing a positive relationship between phenomenal quality and target duration, and some showing a negative or U-shaped relationship. On average, judgements of duration quality increased with target duration, whereas no such increase was observed for POT quality. Similarly, duration quality was judged as being better for auditory than for visual stimuli, but no such modality effect was observed for POT quality. Participants with a positive relationship between POT quality and target duration, however, also showed better POT quality for auditory than for visual stimuli. Overall, it thus seems that modality not only affects temporal judgements but also their phenomenal quality. Two further aspects of the quality judgement results seem noteworthy. Although it was not possible to clearly determine the thresholds of when an experience of duration and POT arises, the positive quality functions start at a lower level for POT quality, and the negative and positive functions converge for longer durations for duration quality but not for POT quality (see Fig. 4). This suggests that the experience of POT may arise somewhat later than the experience of duration (see Droit-Volet et al., 2017). Additionally, the phenomenal quality of POT is likely to be less pronounced than that of duration in situations such as the present study where no substantial self-conscious affective state (e.g., boredom, feeling happy) is elicited (see, e.g., Martinelli & Droit-Volet, 2022a). Overall, it seems that different participants conceptualized phenomenal quality differently. It is, for example, conceivable that some participants judged the POT quality as being better for relatively shorter target durations because these stimuli disappeared very quickly. Similarly, some participants might have judged duration quality as being better for the shortest and longest durations, as these durations were the most salient within the tested duration range. Clearly, further research is needed to investigate the phenomenal quality of duration and POT experience across different time ranges and identify the causes of interindividual differences in these phenomenal quality judgements.

Previous results suggest that attention (to time) may be an important factor in how the POT is experienced (e.g., Droit-Volet & Wearden, 2015; Martinelli & Droit-Volet, 2022b). In the study by Martinelli and Droit-Volet (2022b), a negative relationship was observed between attention to time and POT judgements, that is, the more participants attended to time, the slower time seemed to pass. At the same time, attention to time was positively related to duration judgements, that is, the more participants attended to time, the longer time was perceived to be. The latter result is consistent with the assumption of internal clock models that an attentional gate regulates the throughput of ticks from a pacemaker to an accumulator that counts the ticks (e.g. Zakay & Block, 1995). However, the effect of modality on perceived duration, as in the present study, is usually attributed to variations in pacemaker speed rather than to differences in attention allocation (e.g., Bratzke & Ulrich, 2019; Wearden et al., 1998). Nevertheless, it is also conceivable that the modality effect on perceived duration is due to a so-called “flickering” switch mode (Lejeune, 1998). Accordingly, a switch that is closed at the beginning of an event to be estimated and opened at the end, may “flicker” for visual but not (or to a lesser degree) for auditory stimuli (e.g., because auditory stimuli grab more attention than visual ones), resulting in a higher tick rate for auditory than for visual stimuli (see Penney et al., 2000). However, it is unclear how such potential differences in pacemaker speed or tick rate might be functionally related to the experience of the POT (see also Wearden, 2015). Ultimately, the role of attention in the modality effects observed in the present study is difficult to assess. In order to gain insight into the role of attention in this context, it would be useful in future studies to also ask how much attention the participants paid to time.

One might be inclined to assume that in order to provide a POT judgement participants simply inverted their duration judgement, implying that POT and duration judgements reflect the same (duration) judgement. Although this possibility cannot be completely ruled out, it seems rather unlikely for several reasons. First, as already mentioned in the *Introduction*, previous research has observed empirical dissociations between the two different types of judgements (e.g., Droit-Volet & Wearden, 2016; Martinelli & Droit-Volet, 2022a). Second, although duration and POT judgements showed moderate to strong correlations in the present study, the relationship between duration and POT quality judgements was much less pronounced. If POT judgements were simply inversions of duration judgements, one would probably also expect high correlations between the two quality judgements. Alternatively, participants could have inferred their POT judgements from the duration judgements because they did not (or only to a very small degree) experience a POT. In this case, one would expect generally low POT quality judgements, which was also not observed. Finally, the results of the phenomenal quality judgements suggested that the experience of POT might arise somewhat later than the experience of duration. However, this result requires further investigation, as the difference was rather subtle and the quality judgements showed great variability.

Another related possible limitation of the present study is a response bias, due to the presentation of the four VASs on the same screen in the same presentation order, and the same spatial nature of the VAS for the different judgements, which might induce a tendency to provide consistent responses across the different VASs. Such a response bias, however, seems unlikely, as all participants showed a positive relationship between target duration and duration judgements, whereas other measures showed interindividual differences in the direction of the relationship with target duration. Nevertheless, future studies could attempt to reduce the potential for such a response bias using different timing methods for duration and POT judgements. Another potential limitation is the online format, which did not allow preventing interruptions or controlling other confounding variables during the experiment. However, the influence of such uncontrolled variables seems rather small, as all participants showed an effect of target duration on duration judgements, and the previously observed interaction between target duration and modality on duration judgements (e.g., Wearden et al., 1998) was replicated.

In conclusion, modality differences in time perception seem to occur not only in duration but also in POT judgements. Specifically, more time seems to pass more slowly with auditory than with visual stimuli, at least in the milliseconds to seconds range. Whether the “more time” is a consequence of the slower POT or vice versa, or whether these two effects reflect two sides of the same coin, remains an open question.

## Data Availability

The datasets generated during and/or analysed during the current study are available via the OSF at https://osf.io/5uda4/
