# Correction to: Do “auditory” and “visual” time really feel the same? Effects of stimulus modality on duration and passage-of-time judgements

**DOI:** 10.3758/s13414-025-03153-z

**Published:** 2025-08-26

**Authors:** Daniel Bratzke

**Affiliations:** Department of Psychology, University of Bremen, Bremen, Germany

**Correction to: Attention, Perception, & Psychophysics**

10.3758/s13414-025-03131-5

Figure 1 was originally published with grid lines. The grid lines have been removed (otherwise it is identical to the originally published figure).

The original article has been corrected.

